# Myxobacteria as a Source of New Bioactive Compounds: A Perspective Study

**DOI:** 10.3390/pharmaceutics13081265

**Published:** 2021-08-16

**Authors:** Mudasir Ahmad Bhat, Awdhesh Kumar Mishra, Mujtaba Aamir Bhat, Mohammad Iqbal Banday, Ommer Bashir, Irfan A. Rather, Safikur Rahman, Ali Asghar Shah, Arif Tasleem Jan

**Affiliations:** 1 Department of Biotechnology, Baba Ghulam Shah Badshah University, Rajouri 185234, Jammu and Kashmir, India; mudasirbiotech@bgsbu.ac.in; 2 Department of Biotechnology, Yeungnam University, Gyeongsan 38541, Korea; awdhesh@ynu.ac.kr; 3 Department of Botany, Baba Ghulam Shah Badshah University, Rajouri 185234, Jammu and Kashmir, India; mujtaba@bgsbu.ac.in; 4 Department of Microbiology, Baba Ghulam Shah Badshah University, Rajouri 185234, Jammu and Kashmir, India; bandayiqbal.1000@gmail.com; 5 Department of School Education, Jammu 181205, Jammu and Kashmir, India; ommerbashir@gmail.com; 6 Department of Biological Sciences, Faculty of Science, King Abdulaziz University (KAU), Jeddah 21589, Saudi Arabia; ammm@kau.edu.sa; 7 Department of Botany, MS College, BR Ambedkar Bihar University, Muzaffarpur 845401, Bihar, India; shafique2@gmail.com

**Keywords:** antibiotics, bioactive compounds, medication, Myxobacteria, human diseases

## Abstract

Myxobacteria are unicellular, Gram-negative, soil-dwelling, gliding bacteria that belong to class δ-proteobacteria and order *Myxococcales.* They grow and proliferate by transverse fission under normal conditions, but form fruiting bodies which contain myxospores during unfavorable conditions. In view of the escalating problem of antibiotic resistance among disease-causing pathogens, it becomes mandatory to search for new antibiotics effective against such pathogens from natural sources. Among the different approaches, Myxobacteria, having a rich armor of secondary metabolites, preferably derivatives of polyketide synthases (PKSs) along with non-ribosomal peptide synthases (NRPSs) and their hybrids, are currently being explored as producers of new antibiotics. The *Myxobacterial* species are functionally characterized to assess their ability to produce antibacterial, antifungal, anticancer, antimalarial, immunosuppressive, cytotoxic and antioxidative bioactive compounds. In our study, we have found their compounds to be effective against a wide range of pathogens associated with the concurrence of different infectious diseases.

## 1. Introduction

Myxobacteria, bacteria belonging to family δ-proteobacteria and order Myxococcales, are unicellular, soil-dwelling, rod-shaped bacteria that display gliding motility on attachment to solid surfaces. They are omnipresent, with habitats ranging from tundra to hot deserts and from acidic soils to alkaline conditions [1,2,3]. The source for their isolation ranges from soil to decaying wood and leaves of trees up to excreta of herbivorous creatures [4,5]. Under nutrient-deficient conditions, they produce species-explicit structures (fruiting bodies) that exhibit myxospores (arisen from vegetative cells) within themselves to pass decades of unfavorable environmental conditions [6]. Withstanding regular confinement endeavors, myxospores sprout with the onset of favorable conditions into full-fledged structures, with the exception of depicted facultative anaerobic species, *Anaeromyxobacter dehalogenans* [7]. Recently, a large number of studies have been performed to gain a detailed account of the *Myxobacterial* properties along with types, dynamics and biogenesis of Myxobacteria-derived secondary metabolites [8,9,10,11,12].

The rise in resistance to armor of available antibiotic regimes represents a problem of global magnitude [13,14,15,16]. With increases in mortality and morbidity rates, it becomes imperative to have a strategic management plan to monitor the impact of resistance development and means for exploration of new molecules that can combat the emergence of different diseases among humans [12,17]. *Myxobacterial* species, despite exhibiting sensitiveness to tetracycline, kanamycin, erythromycin, streptomycin, neomycin and actinomycin, produce a variety of chemically different structures that in due course were found effective in combatting the growing problem of drug resistance. The present study highlights the potential of Myxobacteria as a source of new bioactive molecules, with strong emphasis on the production and screening of secondary metabolites, their effect observed in overcoming the odyssey associated with different diseases, as well as having updated information of the current development of their exploitation as a source of effective molecules with potential to compliment available drugs in the control of different diseases.

## 2. Distribution

Myxobacteria are largely cosmopolitan. Besides inhabiting terrestrial conditions, they mark their presence in extreme habitats, such as anaerobic/microaerophilic, freshwater, acidic soils, saline waters and others [12]. Since maximum populations of Myxobacteria predominantly inhabit terrestrial ecosystems, a large proportion of their secretions (secondary metabolites) are derived from terrestrial *Myxobacterial* species. On the basis of habitats, their distribution is studied under the following.

### 2.1. Terrestrial Habitats

Adaptation of Myxobacteria to terrestrial habitats manifests their existence in wide phenotypic characteristics, such as social swarming and gliding, resting myxospores, etc., capable of producing secondary metabolites with a wide range of antibiotic or antifungal activity as well as predation or cellulose decomposition [18]. With the help of different probes and primers, Wu et al. explored a wide range of Myxobacteria, mostly Myxococcales, from the soil samples [19]. Mohr revealed greater presentation of *Myxococcus* and *Corallococcus* genera by standardized cultivation techniques as compared to cultivation-independent clone libraries [12].

### 2.2. Acidic and Alkaline Habitats

Generally, Myxobacteria inhabit the soils which are neutral or slightly alkaline and show a narrow range in their pH, i.e., approximately 6.5–8.5 [12]. Myxobacteria species isolated from the alkaline bogs include *Myxococcus*, *Archangium* and *Sorangium,* along with others such as *Melittangium* [20]. *Corallococcus coralloides* (formerly *Myxococcus coralloides*) dominated in slightly acidic soils, while *M. fulvus* dominated in soils with a pH range in between 3.0 and 3.5 [21]. Ruckert reported that *Myxobacterial* diversity decreases with the decrease in the pH of the soil at alpine regions [21].

### 2.3. Freshwater Habitats

Freshwater-dwelling Myxobacteria share some characteristic features with soil inhabitants, which justifies that these Myxobacteria have been blown away or washed from soil into the freshwater bodies [22]. Research related to freshwater habitats of Myxobacteria reveal that in lake mud, Myxobacteria were the dominant bacterial groups [23].

### 2.4. Marine/Saline Environments

Though Myxobacteria are less adapted to saline environments, their existence in salty conditions was reported by Brockman in 1963, who observed *Myxobacterial* fruiting bodies in sand dunes from an ocean beach of South Carolina [24]. Marine Myxobacteria are represented by four different genera: Salimabromide [25], Enhygrolides [26], Haliangicin [27] and Haliamide [28]. *Haliangium tepidum* and *H. ochraceum* are the representative members of Myxobacteria from coastal salt marshes. They differ from members of the terrestrial genus with respect to the presence of anteiso-branched fatty acids, that help them to survive in greater salt concentrations (2–3% NaCl) [29]. Some genera of Myxobacteria, including Enhygromyxa [30], Plesiocystis [31] and Pseudenhygromyxa [32], are entirely detected in the saline environments. Brinkhoff et al. reported a cluster of marine Myxobacteria (MMB) from sediments of the North Sea [33,34]. Zhang et al. studied 58 species of Myxobacteria from the saline soils of Xinjiang, China [35], and Li et al. observed that species such as *Sorangium*, *Cystobacter*, *Myxococcus*, *Polyangium*, *Corallococcus* and *Nannocystis* show better survival in elevated salt conditions [36].

### 2.5. Facultative Anaerobic Myxobacteria

Myxobacteria are strictly aerobes, with the exception of *Anaeromyxobacter dehalogenans*, which is a facultative anaerobe. This strain of Myxobacteria was studied from sediments of the stream and grows with 2-chlorophenol (2-CPh) as an electron acceptor and acetate as an electron donor [7]. Later, different strains of this Myxobacteria were isolated from uranium-contaminated soils [37], flooded paddy fields [38], corrosive material of water pipelines [39] and arsenic-polluted environments [40].

### 2.6. Myxobacteria Inhabiting Moderate to Extreme Environments

Most of the *Myxobacterial* species are mesophilic, i.e., they survive in the range of 4–44 °C. However, they are also reported to survive in the extreme temperature range. Myxospores liberated by bacteria inhabiting extreme environments act as a means of sexual reproduction and can survive with temperature extremes of 58–60 °C. Production of myxospores differentiates these organisms from the rest of the faunal diversity [22]. Brockman analyzed greater diversity among Myxobacteria from regions that received greater annual rainfall (400–800 mm) as compared to the normal range of 200–400 mm [41]. Gerth and Müller [42] reported that Cystobacterineae and Sorangiineae-*Myxobacterial* suborders show greater morphogenesis at temperatures of 42–48 °C. Mohr et al. reported that *N. konarekensis*, which was studied from an Iranian desert, exhibits the best growth at 37 °C, compared with *N. pusilla* and *N. exedens,* which show optimal growth at 30 °C [43]. Though hot springs are not considered suitable for the growth of mesophilic Myxobacteria, Iizuka et al. reported four different strains of Myxobacteria that grow in geothermal conditions (optimum 45–49 °C) from Japan [44].

## 3. *Myxobacterial* Secondary Metabolites

Secondary metabolites represent incredible gathering of characteristically differing molecules blended among different creatures, such as microorganisms, plants, etc. Though they are not actively involved in development or any type of advancement, their absence prompts a long-haul disability in the survivability of living beings [45]. Production of secondary metabolites has been reported from a large number of *Myxobacterial* species, but a major proportion of them are reported among *Myxococcus xanthus*, *Sorangium cellulosum* and *Chondromyces* species [46]. In addition to ribosomally produced secondary metabolites, a major proportion of *Myxobacterial* metabolites were found to be derivatives of polyketide synthases (PKSs), non-ribosomal peptide synthetases (NRPSs) or hybrids of PK-NRPS systems [3,6,47]. The synthesis module in both cases proceeds through buildup of monomeric blocks: acyl CoA thioester (in case of PK metabolites) and amino acids (both proteinogenic and non-proteinogenic in case of NRPs), in a stepwise manner, followed by modification either during assembly of reaction intermediates or at the end after release from the multienzyme complex [3]. Over the past 3 decades, more than 100 secondary metabolites with over 600 analogs were reportedly isolated from more than 9000 *Myxobacterial* strains [48]. The production of unique metabolites among *Myxobacterial* strains reflects a strong correlation between genome size and the biosynthetic pathway [49,50].

Considered as a rich source of secondary metabolites, the production of a large number (>80 distinctive and 350 structural variants) of bioactive compounds by Myxobacteria puts it on par with *Pseudomonas* for being a rich source of antibiotics [51]. A large number of *Myxobacterial* secondary metabolites show similarity to those produced by *Pseudomonas* and *Bacillus* spp. Antibiotics produced as bioactive secondary metabolites have been observed for about 55% and 95% of *Myxobacterial* spp. that exhibit bacteriolytic and cellulolytic properties [52]. With greater potential for use in clinical settings, compounds isolated from Myxobacteria are found either as macrocyclic lactones or linear cyclic peptides [51,52]. Information on different aspects of secondary metabolites produced by different strains of Myxobacteria along with their uses is summarized in Table 1.

## 4. Pharmacological Effects of Myxobacteria-Derived Bioactive Compounds

Myxobacteria, an adaptable cosmopolitan, produces a wide range of bioactive molecules. About 40% of Myxobacteria-derived compounds represent novel (mostly non-glycosylated) chemical structures that act against targets often not covered by compounds derived from *Actinomycetes*, *Bacillus* and *Pseudomonas*. A variety of bioactive compounds produced by *Myxobacterial* spp. play a vital role in biological activities, and mostly, their activities are antifungal, antibacterial, anti-cancerous, antiparasitic and immunomodulatory.

### 4.1. Myxobacteria and Infectious Diseases

Before the advent of an era of widely accessible anti-infectious agents, mankind was considered vulnerable to infections such as cholera, which reached the extent of epidemics that caused a huge loss of human lives [131]. With the passage of time, the period of anti-infectious agents moved along from quinine (utilized against fever), to Salvarsan (arsenic compound used against syphilis) and Sulpha drugs such as Protonsil (utilized against diseases caused by Gram-positive *cocci*). The circumstances profoundly improved with the discovery of the β-lactam drug Penicillin, from *Penicillium* spp. [132]. The era of antibiotics moved on to aminoglycosides [133], macrolides [134] and so on to treat ailments that were considered untreatable. Inaccurate recommendation and wrong use of antibiotics in human medication, veterinary and horticulture expanded portability, and as such, quick spread of microbes, that raised alarm regarding the use of multi-tranquilize safe microbes. Many pharmaceutical companies withdrew from manufacturing new drugs due to high-cost screening systems developed for nosocomial infections caused by ESKAPE (*Enterococcus faecium*, *Staphylococcus aureus*, *Klebsiella pneumoniae*, *Acinetobacter baumannii*, *Pseudomonas aeruginosa* and *Enterobacter* spp.) pathogens [135]. With less new medications, the dying antimicrobial pipeline caused by an absence in development and inefficient ways of screening bioactive substances presented a dreadful situation that led to obstruction in the production of drugs [136,137]. The bottlenecks that choked the production of anti-infective agents prompted qualified countermeasures to be implemented regarding improvements in the production of engineered medications, proper screening of the metabolite markers, followed by assessment of the rediscovered drugs. At this instance, exploration of new genera and species are of extraordinary intrigue [138] as it may involve the creation of auxiliary metabolites in scaleup forms or fitting hardware for maturation and release of substances from fermenter stock for resolving biotic and abiotic conditions of the maker strain.

Myxobacteria, together with *actinomycetes* [139] and *Bacillus* spp., are considered as the best producers of bioactive compounds [140]. A large proportion of Myxobacteria-derived bioactive compounds (29%) displaying antibacterial properties reflect their competitiveness for existence in their natural habitats. These characteristic products demonstrate a more extensive scope of biological activities which are regularly less direct to rationalize, as the production of regular objects from different *Myxobacterial* spp. requires regular screening and enormous scaleup development [6].

### 4.2. Myxobacteria and Viral Diseases

#### 4.2.1. Human Immunodeficiency Virus (HIV)

Human Immunodeficiency Virus is a single-strand RNA (ssRNA) lentivirus which targets human immune cells, and integrates into host DNA by reverse transcription. Secondary metabolites extracted from different *Myxobacterial* strains are reported to play crucial roles against HIV. The Sulfangolids are an important class of antiviral secondary metabolites secreted by different strains of *Sorangium cellulosum* [105]. *Myxobacterial* extracts such as spirangien B, sulfangolid C, soraphen F and epothilon D at different concentrations showed impressive activity against HIV [124]. Soraphens exert antiviral activity by inhibiting acetyl-CoA carboxylate transferase [141], while epothilones stabilize the activity of macrophage microtubuli in a parallel way to Taxol^®^ [142,143]. Ixabepilone^®^, an FDA-registered anticancer drug, is derived from epothilone B [144]. Epothilon D and spirangien B are believed to decrease the phosphorylation, and as such degradation of inhibitor of kappa B (IkBS) [143,145]. Rhizopodin, a well-known actin inhibitor, extracted from *Myxococcus stipitatus* [124], interferes in virus synapses and hence blocks the virological synapse arrangement. Stigmatellin extracted from *Stiginatella aurantiaca* Sga15, disorazol extracted from *Sorangium cellulosum* Soce 56 and tubulysin extracted from *Archangium gephyrs* strain Ar315 shows mild anti-HIV activity [124], while Phenalamide A1, phenoxan and thiangazole separated from *Polyangium* sp. and *Myxococcus stipitatus* strain Mxs40 suppress HIV-1-mediated cell death in the MT-4 cell assay, thereby exhibiting high anti-HIV activity [146]. Aetheramide A and B isolated from the genus *Aetherobacter,* that inhibits HIV-1 infection, show IC_50_ values of 0.015 and 0.018 M, respectively [109,147,148]. Similarly, Ratjadon A (a compound isolated from *Sorangium cellulosum* Soce 360), capable of blocking the Rev/CRM1-mediated nuclear export, inhibits HIV infectivity; however, its toxicity and low SI value becomes a limiting factor for its exploitation as a potential therapeutic molecule [106,149].

#### 4.2.2. Human Cytomegalovirus (HCMV)

Infections of Human Cytomegalovirus are associated with diseases such as glandular fever and pneumonia. Myxochelin, a secondary metabolite obtained from different *Myxobacterial* strains, responsible for iron uptake during iron-limiting circumstances, was found to be a potent antitumor agent [87,150,151]. The ability of nannochelins and hylachelins (siderophores of *Myxobacterial* source) in inhibiting the human 5-lipoxygenase (5-LO, a gene associated with the proliferation of cancerous cells) were found exerting antitumor activity [87,142,143,144,145,146,147,148,149,150,151,152,153,154]. It is believed that a similar pathway of inhibiting 5-LO is associated with the strong anticancer activity of myxochelin [153,155]. Of the different Myxochelins, which are either isolated from *Angiococcus disciformis* (strain And30) or synthesized [155,156], Myxochelin C is capable of inhibiting HCMV (IC_50_ value of 0.7 g/mL) [150,157]. It opens avenues for testing other known siderophores, such as nannochelins, hylachelins and myxochelin analogues, in the future for their possible role in inhibiting HCMV [158]. Additionally, structure–activity relationships of the siderophores need to be studied for possible discovery of more potent antivirals [123].

#### 4.2.3. Ebola Virus Disease (EVD)

Ebola virus (EBOV) is a single-stranded RNA virus which causes hemorrhagic fever. Different metabolites extracted from Myxobacteria were analyzed for their possible activity in inhibiting the Ebola virus using GP-pseudo-typed lentiviral vectors expressing Ebola envelope glycoprotein [97]. Chondramides extracted from the genus Chondromyces [159] of Myxobacteria were found capable of inhibiting the EBOV-GP-mediated transduction [123]. Noricumazole, a polyketide extracted from *Sorangium cellulosum,* exerts an EBOV-GP inhibitory effect with an IC_50_ value of 0.33 M. [97]. The secondary metabolite is believed to lower the virulence of EBOV via blocking of the potassium channels [76,97].

#### 4.2.4. Hepatitis C Virus (HCV)

Hepatitis C virus, a single-stranded RNA virus, undergoes transmission through blood transfusions. Heterocyclic metabolites such as labindoles A and B [160], 3-chloro-9H-carbazole and 4-hydroxymethyl-quinoline extracted from *Myxobacterial* strain *Labilithrix luteola*, exert potent antiviral activity, and thereby help to overcome the effects of HCV [160]. Of the different macrolactones, Soraphens A obtained from *Myxobacterial* species was found to inhibit HCV replication in in vitro HCV culture models (cells in sub-genomic and full-length replicons) and in cell culture-adapted virus with an IC_50_ value of 5 nM [96,161,162,163]. Lanyamycin, a macrolide obtained from *Sorangium cellulosum* (strain Soce 481) that exhibits similarity to bafilomycins of actinobacteria effective against influenza A virus (IC_50_ value of 0.1 nM), was found to moderately inhibit HCV [96,160,164].

### 4.3. Myxobacterial Metabolites as Anti-Neurodegenerative Diseases

Inside the cell, the endoplasmic reticulum (ER) helps in the processing of proteins before their transport to the target sites. However, any kind of ER dysfunction due to protein misfolding may lead to neurodegenerative disorder or cell death [165,166,167]. *Myxobacterial* secondary metabolites act on protein GRP78/Bip, which helps to release any kind of stress created in the ER [168]. It also decreases the release of apoptosis-inducing factor (AIF) and cytochrome C (an apoptosis-related marker proteins). Therefore, *Myxobacterial* secondary metabolites help in combating the Parkinson’s disease (PD) pathology via decreasing the ER stress, which contributes to inhibition of cell apoptosis [169]. Microtubules play a major role in the axoplasmic transport of different constituents of the cell (mitochondria, synaptic vesicles, lipids, proteins) [170]. Neurodegenerative diseases such as Alzheimer’s disease (AD), Amyotrophic lateral Sclerosis (ALS) and PD arise by distraction in the axoplasmic transport due to microtubules linked to tau proteins—the phenomenon known as tauopathy [171,172,173,174,175]. Epothilones (A–F) are a particular class of secondary metabolites produced by *Sorangium cellulosum* strain So ce90 that exhibit antifungal and anti-cancerous potential [176]. These compounds bind to microtubules and help them in stabilization, hence resulting in the elevation of axoplasmic transport in neurodegenerative disorders [177]. Of the different Epothilones, Epothilone D plays an important role in improving the axonal transport, as well as protecting cognitive deficits in a mouse tauopathy model having overexpression of P301S (a mutant tau), thereby contributing to inhibition of tau pathology [178]. Epothilone D also plays an active role in alleviating the microtubule defects in a C57Bl model of PD [179].

Neurodegenerative diseases such as PD, AD and Huntington’s Disease (HD) are the outcomes of different mitochondrial dysfunctions [180]. Earlier studies predicted that certain prokaryotes have the ability to synthesize PUFAs, however, these predictions failed as some extremophilic bacteria which inhabit extreme environments of seas and oceans invalidated this hypothesis [181,182]. Among different terrestrial prokaryotes, Myxobacteria are considered as a major contributor of PUFAs [183]. In the studies employing the genome mining approach, two *Myxobacterial* species, *Sorangium* and *Aetherobacter*, were found, having different organization of gene clusters associated with biosynthetic PUFA compared with their marine counterparts [184]. *Myxobacterial* omega 3 PUFAs play an antagonistic role against prenatal stress, which arises from mitochondrial abnormalities such as changes in mitochondrial complexes, DNA damage and memory deficiency [185,186]. Having a remarkable effect regarding the phospholipid profile, and as such fluidity of the mitochondrial membrane, DHA was observed to play a critical role in maintaining stability of the structure, and as such functions of the mitochondrial membrane, and thereby in non-amyloidogenic processing of APP in the HEK-APP cell line [187].

#### Immune Modulating *Myxobacterial* Compounds

Employment of *Myxobacterial* secondary metabolites such as Soraphen A, bengamide A and B and Spirangiens as immune-enhancing compounds has attracted the attention of different researchers throughout the world [188]. Castro et al. worked out the immune-enhancing responses of Soraphen A [189]. Acting on the biotin carboxylase (BC) domain, Soraphen A extracted from *Sorangium cellulosum* So ce26 was found to exert an inhibitory effect on acetyl-CoA carboxylase (ACC) [141]. Bengamides, an important class of secondary metabolites produced by *Myxococcus virescens*, exert both anti-inflammatory as well as immune-boosting effects via regulation of the nuclear factor-KB (NF-KB) and pro-inflammatory cytokines (IL-6, TNFα and MCP-1) [190]. Spirangien A produced by *Sorangium cellulosum* strain So ce90 shows antifungal activity, as well as suppressing transcription of IL-8 in response to IL-1 (cytotoxic activity). The compound along with its derivative, spirangien M522, were found effective in inhibiting IL-8 gene expression in the HeLa cell line [145].

### 4.4. Myxobacterial Compounds Attributing Cytotoxic Effects

*Myxobacterial* secondary metabolites display unique structural properties and exhibit novel modes of action. These metabolites mainly target the cellular structures that are rarely hit by metabolites from other sources.

#### 4.4.1. Compounds Targeting Electron Transport

*Myxobacterial* compounds such as crocacins [191] and aurachin C [192,193], along with a group of closely related bithiazole derivatives, particularly myxothiazol, cystothiazol and melithiazole [66,194,195,196], were found effective in inhibiting mitochondrial respiration through interference in the functioning of complex-I (NADH-Ubiquinone oxidoreductase) and complex-III (Cyt b–C1 complex). Stigmatellin was found to exert its inhibitory effect at complex III of the mitochondria [6] and Cyt b6/f of the photosynthetic apparatus in plants [197,198,199].

#### 4.4.2. Compounds Targeting RNA and Protein Synthesis

With enormous potential to lead as building blocks for drug development, compounds of Myxobacteria origin such as saframycin tie to DNA [200], ambruticin helps in osmoregulation of fungi [126] and gephyronic acid [201] and myxovalargin [93,202] repress eukaryotic and prokaryotic protein synthesis, respectively [83]. Etnangien is a metabolite that targets protein synthetic machinery via inhibition of the eubacterial RNA polymerases. In addition to rifampicins utilized maximally in clinics, other inhibitors of RNA polymerase of *Myxobacterial* origin include thiolutin [203,204], streptolydigin [205] and holomycin [206]. These molecules (ripostatin and corallopyronin) show no cross-resistance with rifamycin, and likewise concentrate on the commencement of RNA synthesis [207]. Acting in an alternate way to rifamycin, it is believed that these metabolites can potentially be used to overcome rifamycin resistance in bacteria [208,209]. Inhibition of the protein synthetic machinery is mediated by both naturally occurring compounds such as sorangicins and ripostatins that exert their effect during initiation (sorangicins) [210,211] and chain elongation (ripostatins) [212,213], as well as by chemically related myxopyronins [93] and corallopyronins [214].

Compounds of *Myxobacterial* origin (10% of *Myxobacterial* compounds), that interfere with the microtubule assembly (cytoskeleton) and thereby hinder cell proliferation and promote apoptosis, are currently being used in cancer chemotherapies. Similar to notorious fungal toxins obtained from mushrooms (preferably green and white cap mushrooms), *Myxobacterial* compounds such as rhizopodin [215,216] and chondramides [159,217,218] are reported to work explicitly on the actin [214]. Though all chondramide variants exert similar effects, chondramide C was found to be most effective in its action on actin [217]. Of the different compounds, a few compounds, such as epothilones [219,220], play important roles in retaining tubular polymerization under in vitro conditions, while others, such as tubulysins [221,222], favor depolymerization events of the tubulin. Epothilones and their analogs have shown antitumor activity towards multidrug-resistant and paclitaxel-safe tumor cell lines [223]. In 2007, the FDA recommended Ixabepilone (IxempraTM)—a derivative of epothilone—for the treatment of metastatic breast cancer, while epothilones B and D are currently undergoing clinical trials [224]. From the tubulysins class, tubulysin D displays action that surpasses other tubulin modifiers, such as taxol, epothilones and vinblastine, by 20–100-fold [225,226]. Additionally, tubulysin A is currently explored for its pharmacological properties related to its use as an antiangiogenic and antiproliferative agent [227].

#### 4.4.3. Other Activities

Soraphen A from *Sorangium cellulosum* was found to hinder normal functioning of acetyl-CoA carboxylase through interference with its biotin carboxylase (BC) domain. With its novel modus operandi, Soraphen A has explicit utility as a promising therapeutic (novel inhibitor of ACCs) in the treatment of cancers [3,228]. Its utility as a potent inhibitor in cancers hindered by its poor water solubility and less bioavailability is overruled through generation of either structural variants of this metabolite or through the genetic engineering approach, upholding its bioactivity.

### 4.5. Myxobacteria and Plant Diseases of Bacterial and Fungal Origin

Although the contribution of Myxobacteria to plant health remains largely unexplored, studies have assessed the role of *Myxobacterial* secondary metabolites in the predation of microorganisms and other plant pathogens. Based on their ability to degrade biomolecules, two groups of *Myxobacterial* spp., i.e., bacteriolytic and cellulolytic, have been formed [229]. The Myxobacteria of the bacteriolytic category produce a large number of agriculturally important compounds such as pyrrolnitrin, a thiangazoletic that acts as an antagonistic in the control of phytopathogens that destroy crops [230]. Pyrrolnitrin produced by *Myxobacterial* spp. (*Myxococcus fulvus*, *Cystobacter ferrugineus* and *Corallococcus exiguous*) was found effective in controlling the damping-off of diseases of cotton caused by *Rhizoctonia solani* [229,230]. The ability of Myxobacteria to utilize cellulose categorizes them into two groups: Group I, capable of utilizing inorganic nitrogen compounds during their growth on cellulose and glucose sources (members of the Sorangineae suborder), and Group II, unable to make direct use of cellulose (majority of *Myxobacterial* spp.) and as such, dependent on enzymatic degradative products of proteins (peptides and amino acids) as their source of nitrogen [230]. Under natural conditions, Group II *Myxobacterial* spp. causes lysis of other organisms, such as eubacteria, via secretion of exoenzymes (proteases, lipases, xylanases, etc.). The lysate generated thereof is used as a nutrient by these *Myxobacterial* spp., and tags them with the name “micro-predators” [231], *Myxobacterial* proteolytic enzymes exhibiting both cellulolytic (genus *Sorangium*) and predatory roles (genus *Myxococcus*). These proteases are believed to perform lysis of prey, cellular membrane disruption for cytoplasmic content release and protein hydrolysis for supplying amino acids to the Myxobacteria-like functions [232]. Lipids containing fatty acids c16:1ω5c, utilized along with proteins as an energy and carbon source during the growth of myxobacteria, play pivotal roles in the predation by acting as chemo-attractants for the prey. In *Myxococcus xanthus*, lipolytic enzymes belonging to three families—α/β hydrolases, patatin and GDSL lipases—disintegrate the membrane barrier, thereby releasing fatty acids and cytoplasmic contents of the prey. Genus *Polyangium* was found perforating, and as such lysing, the conidia of *Cochliobolus miyabeanus* and hyphae of *R. solani*. Genus *Sorangium* reduces damping-off of conifers in addition to lysis of microorganisms under culture conditions [231,232]. Additionally, the production of unsaturated fats by *Myxoxoccus xanthus* was found to exert an inhibitory effect on the growth of Fusarium roseum [233]. Taken together, the production of agriculturally important compounds along with a series of lytic enzymes show that Myxobacteria have potential for use as biocontrol agents.

## 5. Techniques for Exploring *Myxobacterial* Metabolites

As emerging endeavors of whole-genome sequencing together with metabolic profiling of *Myxobacterial* species revealed high profundity of secondary metabolites, it becomes necessary to have information on mining genomes of both terrestrial and marine Myxobacteria for novel metabolites [234]. It becomes obligatory to have a strategic plan regarding the methodology (in terms of media composition, temperature, pH, along with others) adopted for identification of secondary metabolites from cultivated strains under standard research laboratory conditions. One such strategy is OSMAC (one strain many compounds), initially introduced in Actinomycetes and fungi during isolation of new secondary metabolites [235]. Traditional but untested strategies for isolation of secondary metabolites include inoculation of microorganisms into the culture, much like induction of cytotoxic compounds [236].

Optimization of the growth conditions along with addition of the explicit precursors would be a way to support generation and expansion of the metabolite yield [234]. The adoption of the genetic engineering techniques for producing a strain with desired characters can be achieved. For instance, overexpression of a particular gene activator regulating biosynthesis of a cryptic gene cluster might be activated, as recently illustrated for the fungus *Aspergillus nidulans* [237]. The irregular transposon mutagenesis approach was adopted to obtain genetic information regarding gene clusters of metabolites produced from a prepared cosmid library of the strain [238]. The methodology helped in obtaining information of the gene clusters for ambruticin/jerangolid [239,240], aurachin [240,241], disorazol [242] and tubulysin [243] metabolites. In *Cystobacter fuscus* Cb f17, irregular transposon mutation helped in the recognition of a particular regulatory element for a metabolite [244]. The adopted methodology helped in unravelling information of the biosynthetic gene cluster with two components (StiR) associated with the synthesis of the polyketide stigmatellin. Recognition of ChiR protein following detachment of the promoter binding protein by the biomagnetic bead assay revealed its role in the biosynthesis of the metabolite chivosazol in *Sorangium cellulosum* So ce56, as its overexpression led to a 5-fold increase in the production of chivosazol [245]. Alternatively, intentional inactivation of the gene cluster followed by screening of mutants for non-production of the explicit metabolite compared with the wild phenotypes helped in the recognition of myxochelins, myxochromides and aurafurones [246,247]. Additionally, shot-gun genome sequencing can be adopted to obtain information of the gene clusters for the identification of different metabolites, as observed for phosphoglycolipid moenomycin A [248,249].

To overcome the problem of recalcitrance of a strain for manipulation, heterologous expression of gene clusters (both orphan and known) in a suitable host that offers advantages for genetic manipulation seems a suitable alternative for exploring the function of genes [247]. Using specific hosts such as *Myxococcus xanthus* and a few other bacterial strains such as *Pseudomonas*, it is possible to arrange different gene sets in a codon-optimized manner for heterologous expression that abolishes the requirement for genetic engineering of the host [250]. Though *Myxococcus xanthus* shares codon usage and other physiological parameters with a majority of the *Myxobacterial* species, *Pseudomonads* offers the advantage of a growth rate on par with *E. coli*, with plasmids harboring inducible promoters. Examples of heterologous expression of gene clusters for metabolites, such as epothilone in *M. Xanthus* [251], *Streptomyces coelicolor* [252] and *E. coli* [253], myxochromide S in *Pseudomonas putida* [247,254], soraphen in *Streptomyces lividans* [255], myxothiazol in both *M. Xanthus* [256] and *P. putida* [257] and flaviolin in three *Pseudomonas* strains [257], are available. Employment of Red/ET recombination technology has overcome the limitation of cluster reconstruction associated with the heterologous gene expression by enabling reconstruction of gene clusters onto a suitable vector [258]. Recently, an approach of combining *Myxobacterial* biosynthetic machineries has been explored for production of novel metabolites in a so-called combinatorial biosynthesis approach [259].

## 6. Conclusions and Future Perspectives

The escalating problem of resistance against the current regime of antibiotics has increased concern, particularly related to treatment of human diseases. It has resulted in a community crisis, necessitating the requirement to undertake studies towards development of effective alternatives that could replace or supplement the antibiotics in counteracting occurrence at a global scale. Based on this scenario, studies were undertaken to explore natural resources towards the development of potent products that offer promise for treatment of different diseases. Exhibiting potent antimicrobial activity, secondary metabolites of microbial origin (in particular Myxobacteria) were investigated for possible use in the prevention and treatment of diseases. Myxobacteria, a highly adaptable and cosmopolitan group of microorganisms, were screened at genome and metabolome levels for identification and characterization of metabolites that can serve as potent lead structures for drug development. Evaluation of the rich repertoire of *Myxobacterial* metabolites for safety, specificity, distribution, immune modulation and anti-infectivity potential revealed information of novel antimicrobials that offer great potential to be utilized in the manufacturing of drugs. Despite the fact that *Myxobacterials* exhibit survival under different habitats and extreme climatic conditions, secondary metabolites of *Myxobacterial* origin were found effective in the treatment of a wide range of diseases. Studies need to be undertaken to gain insight into the production mechanism that holds promise in elucidating the regulatory circuit of different secondary metabolites towards optimal design of a strategic plan for enhancing their production. Alongside strategic approaches for elucidating the potency of the secondary metabolites using recently developed techniques that offer flexibility to approval strategies, consistency in safety, efficacy and delivery methods need to be adapted to broaden exploration, and as such adoption of the secondary metabolites of *Myxobacterial* origin.

## Figures and Tables

**Table 1:** Categorization of *Myxobacterial*-derived secondary metabolites based on their function.

Bioactive Compound	Chemical Structure	Classification	*Myxobacterial* sp.	Uses	References
Bioactive compounds exerting antimicrobial effect					
Ajudazol	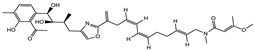	Depsipeptides	*Chondromyces crocatus*	Acts as inhibitor of mitochondrial electron transport. More effective against yeast and fungi.	[3,47,53]
Althiomycin	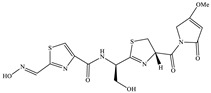	Polyketide, peptide	*Myxococcus xanthus*	Disrupts translocation of tRNA for peptide bond formation by peptidyltransferase. It is effective in treatment of injury and sepsis associated with *Yersinia pestis* infection.	[54]
Angiolactone	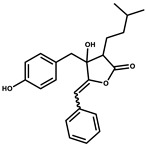	Furanone	*Angiococcus* sp.	Exhibits siderophore production which enables its antibacterial and antiproliferative activity.	[55]
Antalid	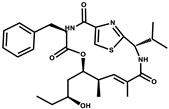	Depsipeptide	*Polyangium* sp.	NA	[56]
Aurachins E	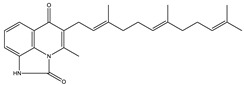	Quinoline alkaloids	*Stigmatella aurantiaca*	Exhibits antimalarial activity (effective against *Plasmodium falciparum*).	[57]
Carolacton	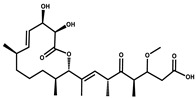	Macrolactone	*Sorangium cellulosum*	Effective in regulating the growth of biofilm-producing microbes such as *Streptococcus mutans* and *pneumococci*.	[58]
Chlorotonil	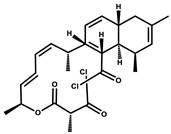	Macrolactone	*Sorangium cellulosum*	Antibacterial and antimalarial activity.	[59]
Corallopyronin A	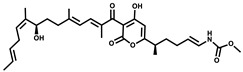	α-Pyrone	*Corallococcus (Myxococcus) coralloides*	Exhibits antibacterial action, effective in treating filariasis.	[60]
Corallorazine	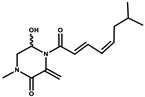	Piperazine	*Corallococcus coralloides*	Exhibits antibacterial activity.	[61]
Crocacin	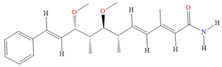	Depsipeptides	*Chondromyces crocatus*	Antibacterial. Inhibits electron transport system.	[62]
Cystobactamid	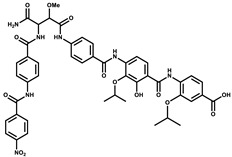	Peptide	*Cystobacter* sp.	Broad-spectrum antibacterial; topoisomerase (gyrase) inhibition.	[63]
Cytochromone	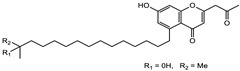	Polyketide, chromone	*Proteus mirabilis*	Essential in mitochondrial electron transport and intrinsic type II apoptosis.	[64]
Cystomanamide	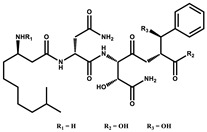	Lipopeptide	*Cystobacter fuscus*	Exhibits strong antifungal and antibacterial activity.	[65]
Cystothiazol	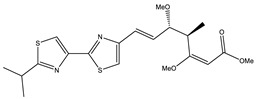	Heterocyclic alkaloid	*Cystobacter fuscus*	Antifungal/cytostatic. Inhibits sub-mitochondrial NADH oxidation.	[66]
Disciformycin	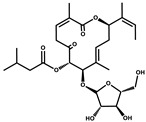	Macrolide	*Pyxidicoccus fallax*	Exhibits antibacterial activity.	[67]
Enhygrolide A	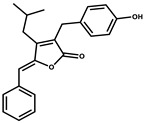	Furanone	*Enhygromyxa salina*	Effective in inhibiting the growth of *Arthrobacter crystallopoietes.*	[68]
Etnangien	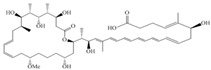	Polyketides	*Sorangium cellulosum*	Works as an inhibitor of eubacterial DNA polymerase.	[3,47,53]
Gulmirecin	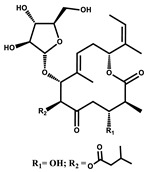	Macrolide	*Pyxidicoccus fallax*	Exhibits antibacterial activity.	[69]
Haliangicin	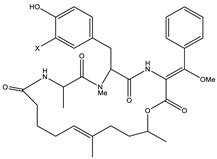	Polyketide	*Haliangium luteum*	Effective against fungi *Aspergillus niger* and *Fusarium* sp. at very low concentrations of 6–12 µg/mL.	[70]
Hyalachelin	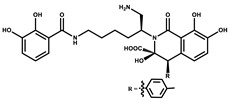	Catechol	*Hyalangium minutum*	Shows sidrophore, i.e., iron-chelating activity, and cytotoxic activity is minor.	[71]
Hyaladione	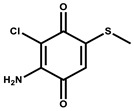	Quinone	*Hyalangium minutum*	Exhibits antimicrobial and cytotoxic activity.	[72]
Hyapyrroline	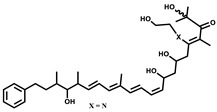	Polyketide, pyrrole	*Hyalangium minutum*	NA	[73]
Hyapyrone	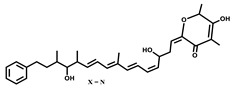	Polyketide, pyrone	*Hyalangium minutum*	Exhibits weak antibacterial and antifungal activity.	[73]
p-Hydroxyacetophenone amide	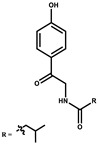	Amide	*Cystobacter ferrugineus*	Shows marginal activity against microalgae (P. simplex).	[74]
1-Hydroxyphenazin-6-yl-a-Darabinofuranoside	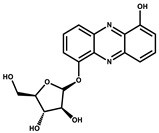	Glycoside	*Nannocystis pusilla*	Exhibits weak antimicrobial activity.	[75]
Icumazol	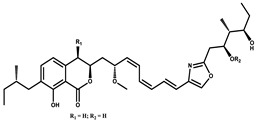	Polyketide	*Sorangium cellulosum*	Effective antifungal. Inhibition of NADH oxidation.	[76]
Indiacen	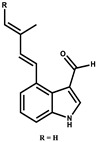	Indole	*Sandaracinus amylolyticus*	Exhibits antibacterial and antifungal activity.	[77,78]
Indothiazinone	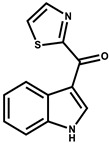	Indole	*Ohtaekwangia kribbensis*	Weak antimicrobial and cytotoxic activity.	[75]
Kulkenon	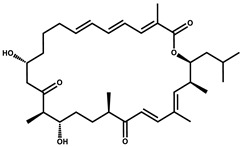	Macrolactone	*Sorangium cellulosum*	Exhibits antibacterial activity.	[79]
Leupyrrins	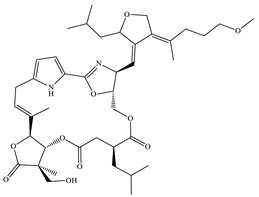	Macrolides	*Sorangium cellulosum*	Exhibits antibacterial activity.	[80]
Macyranone	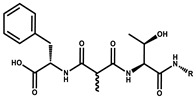	Peptide	*Cystobacter fuscus*	Shows moderate cytotoxic activity; antiparasitic (*L. donovani*); proteasome inhibitor (CT-L activity).	[81]
Maltepolid	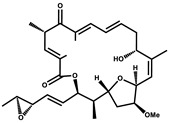	Macrolactone		Exhibits moderate cytotoxic activity.	[82]
Methyl indole-3-carboxylate	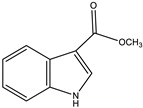	Indole	*Sorangium cellulosum*	NA	[75]
Melithiazols	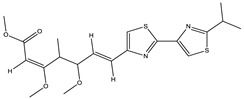	Heterocyclic alkaloid	*Archangium gephyra*	Antibacterial. Inhibits NADH oxidation.	[83]
Microsclerodermin	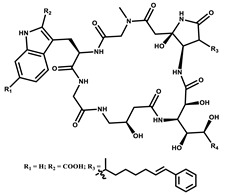	Cyclic peptide	*Microscleroderma, theonella*	Exhibits antifungal activity, NF-kB inhibition and induction of apoptosis.	[84,85]
Myxalamids	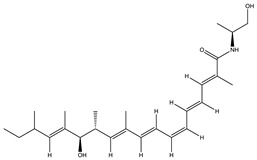	Amide	*Myxococcus xanthus*	Exhibits antibacterial and antifungal activity; inhibits electron transport system.	[3,47,53]
Myxochelin	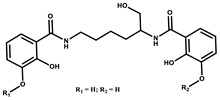	Peptide	*Angiococcus disciformis*	Shows siderophore production. Exhibits antibacterial, antitumor and antiproliferative activities: inhibits 5-lipoxygenase.	[86,87]
Myxocoumarin	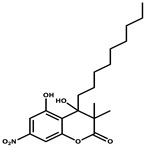	Coumarin	*Stigmatella aurantiaca*	Exhibits antifungal activity.	[88]
Myxoprincomide	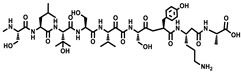	Peptide	*Myxococcus xanthus*	NA	[89,90,91]
Myxopyronin B	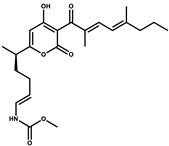	Peptide	*Myxococcus fulvus*	Effective in combating diseases caused by *Staphylococcus aureus*.	[92]
Myxothiazol	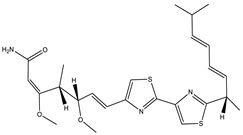	Macrocyclic	*Myxococcus fulvus*	Inhibits mitochondrial cytochrome c reductase.	[3,47,53]
Myxovalargin	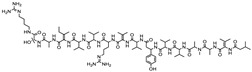	Lipopeptide	*Myxococcus fulvus*	Exhibits antibacterial activity against *Micrococcus luteus* and *Corynebacterium Mediolanum*. Disrupts memebrane integrity and aminoacyl-tRNA binding to site A during translation.	[93]
Myxovirescin	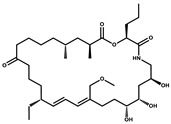	Macrocyclic	*Myxococcus xanthus*	Exhibits antibacterial activity. Blocks bacterial cell wall synthesis via interference in lipid-disaccharide pentapeptide polymerization, as well as targeting type II signal peptidase LspA.	[94,95]
Nannozinone	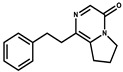	Pyrrolopyrazinoe	*Nannocystis pusilla*	Exhibits weak antimicrobial and cytotoxic activity.	[75]
Noricumazol	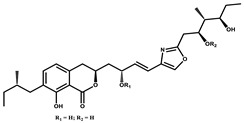	Polyketide	*Sorangium cellulosum*	Inhibits conductance of potassium channel KscA. Exhibits antiviral (EBOV, HCV) activity.	[76,96,97]
Phenoxan	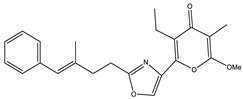	Lipopeptide	*Polyganium* sp.	Effective as an inhibitor of eukaryotic respiratory chain (blocks Complex I). Exhibits antifungal activity.	[3,47,53]
Phoxalone	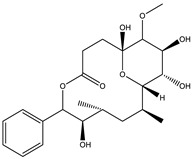	Macrolides	*Sorangium cellulosum*	Exhibits antimicrobial activity.	[98]
Pyrronazol	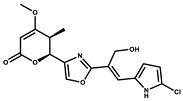	Pyrrole	*Nannocystis pusilla*	Shows weak antifungal activity.	[75]
Ripostatin B	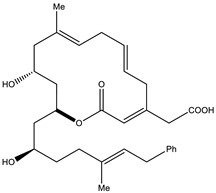	Polyketide	*Sorangium cellulosum*	Effective in treating tuberculosis.	[99]
Roimatacene	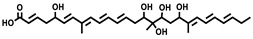	Cyclic peptide	*Cystobacter ferrugineus*	Exhibits antibacterial activity.	[74]
Saframycin Mx1	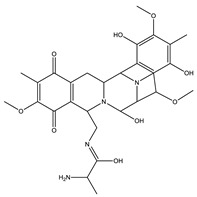	α-cyanoamine	*Myxococcus xanthus*	Acts as a broad-spectrum inhibitor for a wide range of Gram-positive and halobacteria. Shows week activity against Gram-negative bacteria.	[3,47,53]
Salimyxin A and Salimabromide	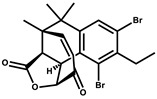 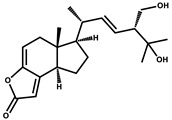	Sterol, Furano lactone	*Enhygromyxa salina*	Effective against *Arthrobacter cristallopoietes.*	[100]
Sesqiterpene	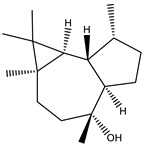	Terpenes	*Sorangium cellulosum*	Exhibits antimicrobial activity.	[101,102]
Sorangicin	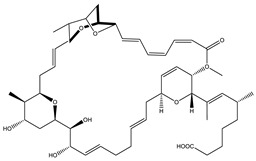	Macrolides	*Sorangium cellulosum*	Exhibits antimicrobial activity.	[82]
Sorangiadenosine	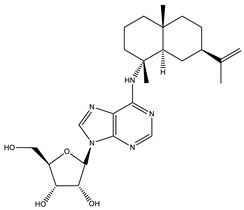	Macrolides	*Sorangium cellulosum*	Exhibits antimicrobial activity.	[101]
Soraphinol	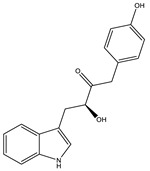	Macrolides	*Sorangium cellulosum*	Exhibits antimicrobial activity.	[103]
Sorazinnone	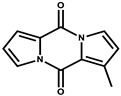	Pyrazinone	*Pyxidicoccus fallax*	Siderophore production. Exhibits antibacterial activity.	[75]
Sorazolon	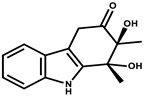	Indole	*Sorangium cellulosum*	Weak activity against Gram-positive bacteria.	[104]
Stigmatellin	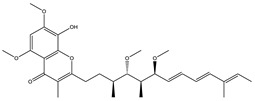	Macrolactone	*Stigmatella aurantica*	Exhibits strong antifungal activity. Inhibits quinol oxidation of mitochondrial cytochrome bc1 complex.	[3,47,53]
Sulfangolid	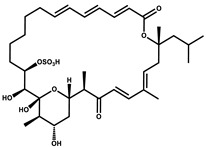	Macrolactone	*Sorangium cellulosum*	Exhibits antiviral (HIV-1) activity.	[105,106]
Thuggacin	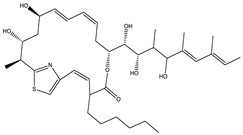	Macrolactone	*Sorangium cellulosum*	Effective against *Mycobacterium tuberculosis*.	[107,108]
	Bioactive compounds exerting cytotoxic effects				
Aetheramide	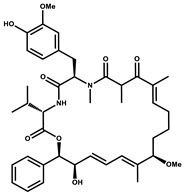	Cyclic peptide	*Atherobacter rufus*	Shows cytotoxic and moderate antifungal activity.	[109,110,111]
Archazolid	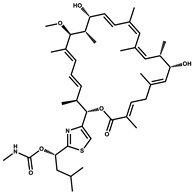	Macrolactone	*Archangium gephyra*, *Cystobacter violaceus*	Exhibits cytotoxic and antitumor activity. Inhibits V-ATPase.	[112]
Argyrin	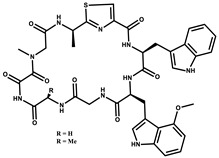	Peptolide	*Archangium gephyra*	Acts as a potential inhibitor of antibody formation by murine B-cells. Exhibits antibacterial and cytotoxic activity.	[113]
Bengamide	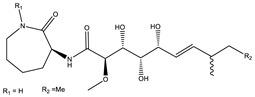	Caprolactam	*Myxococcus virescens*	Shows cytotoxic, antitumor, antibacterial and anthelmintic activity. Inhibits MetAP. Acts as an anti-inflammatory.	[114]
Chivosazol	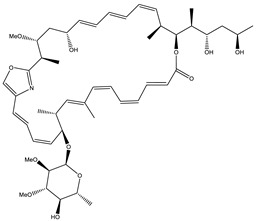	Peptide	*Sorangium cellulosum*	Effective antifungal activity at higher concentration. Exhibits strong cytotoxic activity. Destroys cyto-skeleton.	[3,47,53]
Chrondramide	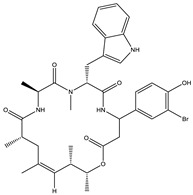	Depsipeptide	*Chondromyces crocatus*	Exhibits strong cytotoxic activity; effective against breast cancer metastasis.	[3,47,53]
Cystodienoic acid	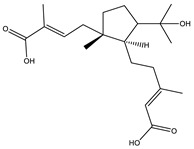	Terpene	*Cystobacter ferrugineus*	Exhibits cytotoxic activity.	[115]
Disorazol	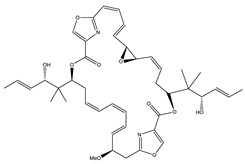	Peptide	*Sorangium cellulosum*	Exhibits strong antifungal activity; inhibits proliferation of different cancer cell lines.	[116]
Eliamid	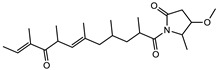	polyketide	*Sorangium cellulosum*	Exhibits cytotoxic activity; shows moderate anthelmintic and antifungal activity; acts as a respiratory chain inhibitor.	[117]
Epothilone	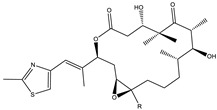	Peptide	*Sorangium cellulosum*	Acts as an inhibitor of microtubule function concerning cell division.	[118]
Haliamide	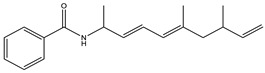	Polyene	*Haliangium ochraceum*	Exhibits moderate cytotoxic activity.	[28]
Hyafurone	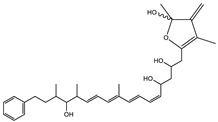	Polyketide, furanone	*Hyalangium minutum*	Exhibits moderate cytotoxic activity, as well as showing marginal antiparasitic activity.	[73]
Miuraenamide	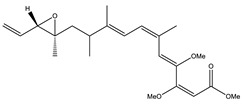	cyclic depsipeptides	*Paraliomyxa miuraensis*	Exhibits antibacterial and cytotoxic activity.	[119]
Nannocystin	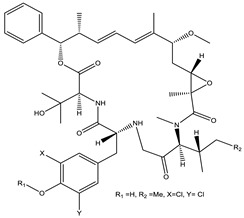	Macrocyclic epoxyamide	*Nannocystis* sp.	Exhibits strong antifungal and cytotoxic activity; inhibits eukaryotic translation elongation factor 1α.	[120,121]
Pellasoren	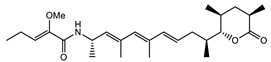	Polyketide	*Sorangium cellulosum*	Exhibits cytotoxic activity.	[51,122]
Ratjadone A	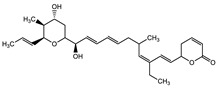	α-pyrone	*Sorangium cellulosum*	Acts as an antiviral drug. Inhibits HIV infection by ceasing the Rev/CRM1-mediated nuclear export.	[106]
Rhizopodin	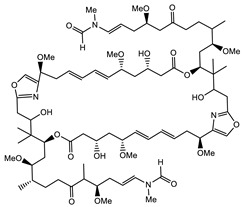	Amide	*Myxococcus stipitatus*	Effective against cancer cell lines. Interferes with cytoskeleton assembly. Acts as a strong antiviral.	[3,123]
Spirangien	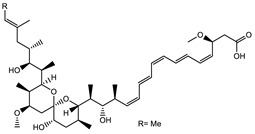	Polyketide	*Sorangium cellulosum*	Exhibits antifungal, cytotoxic, antiviral (HIV) and anti-inflamatory activity.	[124]
Tubulysin	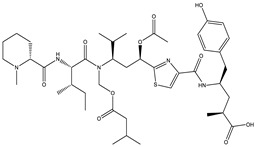	Peptide	*Archangium gephyra* and *Angiococcus disciformis*	It has been found to be effective in treating the cancer associated with Luteinizing Hormone-releasing hormone receptor. Effective in cell cycle arrest at G2/M phase.	[125]
	Bioactive compounds exerting beneficial effects in agriculture				
Ambruticin	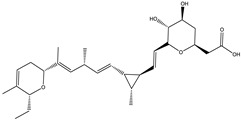	Polyketide	*Sorangium cellulosum*	Acts as a fungicide, effective against *Hansenula anomala* and other plant pathogens such as *Botrytis cinerea,* via interference in osmoregulation system.	[126]
Pyrrolnitrin	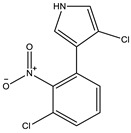	Pyrrole	*Myxococcus fulvus*, *Carallococcus exiguous*, *Cystobacter ferrugineus*	Exhibits strong antifungal activity.	[3,47,53]
Tartrolon	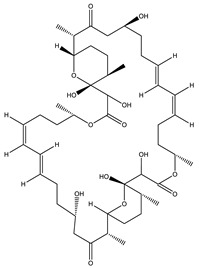	Pilyketide	*Sorangium cellulosum*	Effective against Gram-positive bacteria and mammalian cells.	[127]
Thiangazol	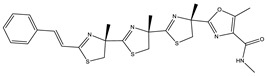	Amide	*Polyangium* sp.	Exhibits strong antifungal, acaricidal and insecticidal activities, as well as having anti-HIV activity.	[128]
Soraphen A	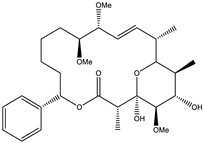	Polyketide	*Sorangium cellulosum*	Effective as a plant disease control agent. Possesses strong antifungal activity. Acts as a broad-spectrum antiviral (effective against HIV and Hepatitis C Virus).	[129,130]

## Data Availability

Not applicable.
